# Eplerenone attenuated cardiac steatosis, apoptosis and diastolic dysfunction in experimental type-II diabetes

**DOI:** 10.1186/1475-2840-12-172

**Published:** 2013-11-21

**Authors:** Elisa Ramírez, Mercedes Klett-Mingo, Sara Ares-Carrasco, Belén Picatoste, Alessia Ferrarini, Francisco J Rupérez, Alicia Caro-Vadillo, Coral Barbas, Jesús Egido, José Tuñón, Óscar Lorenzo

**Affiliations:** 1 Cardiovascular Pathology laboratory, IIS-Fundación Jiménez Díaz, Autónoma University, Av. Reyes Católicos 2, Madrid 28040 Spain; 2 Spanish Biomedical Research Centre in Diabetes and Associated Metabolic Disorders (CIBERDEM) network, Madrid, Spain; 3 Veterinary School, Complutense University, Madrid, Spain; 4 Center for Metabolomics and Bioanalysis (CEMBIO), Facultad de Farmacia, Universidad CEU San Pablo, Campus Montepríncipe, 28668 Madrid, Spain

**Keywords:** Diabetic cardiomyopathy, Eplerenone, Steatosis, Apoptosis

## Abstract

**Background:**

Cardiac steatosis and apoptosis are key processes in diabetic cardiomyopathy, but the underlying mechanisms have not been elucidated, leading to a lack of effective therapy. The mineralocorticoid receptor blocker, eplerenone, has demonstrated anti-fibrotic actions in the diabetic heart. However, its effects on the fatty-acid accumulation and apoptotic responses have not been revealed.

**Methods:**

Non-hypertensive Zucker Diabetic Fatty (ZDF) rats received eplerenone (25 mg/kg) or vehicle. Zucker Lean (ZL) rats were used as control (n = 10, each group). After 16 weeks, cardiac structure and function was examined, and plasma and hearts were isolated for biochemical and histological approaches. Cultured cardiomyocytes were used for *in vitro* assays to determine the direct effects of eplerenone on high fatty acid and high glucose exposed cells.

**Results:**

In contrast to ZL, ZDF rats exhibited hyperglycemia, hyperlipidemia, insulin-resistance, cardiac steatosis and diastolic dysfunction. The ZDF myocardium also showed increased mitochondrial oxidation and apoptosis. Importantly, eplerenone mitigated these events without altering hyperglycemia. In cultured cardiomyocytes, high-concentrations of palmitate stimulated the fatty-acid uptake (in detriment of glucose assimilation), accumulation of lipid metabolites, mitochondrial dysfunction, and apoptosis. Interestingly, fatty-acid uptake, ceramides formation and apoptosis were also significantly ameliorated by eplerenone.

**Conclusions:**

By blocking mineralocorticoid receptors, eplerenone may attenuate cardiac steatosis and apoptosis, and subsequent remodelling and diastolic dysfunction in obese/type-II diabetic rats.

## Introduction

Type-II diabetes (T2DM) is an increasingly prevalent worldwide disease. Heart failure in these patients, even in the absence of vascular disease, is a common asymptomatic pathology known as diabetic cardiomyopathy (DCM). DCM is characterized by myocardial steatosis, apoptosis, and subsequent remodelling fibrosis and hypertrophy [1]. In addition, diverse comorbidities commonly present in diabetes, such as obesity, may accentuate these responses. In particular, overweight patients with T2DM have a significantly higher level of myocardial steatosis preceding and contributing to the early diastolic dysfunction [2]. The excess of circulating free fatty-acid (FFA) may result in increased cardiac FFA uptake, inadequate storage and metabolism, and consequent lipotoxicity by lipid metabolites such as ceramides and reactive oxygen species (ROS) [3]. However, the underlying molecular mechanisms have been poorly investigated, leading to a lack of a diagnostic method and effective therapy. In this sense, a pharmacological blockade of mineralocorticoid receptors (MR) could show potential benefits. MR are activated with equal affinity by aldosterone and glucocorticoids (mainly cortisol and corticosterone) [4]. Among them, aldosterone is a bioactive steroid of the major cardiovascular regulatory system: the renin-angiotensin-aldosterone system (RAAS). Local RAAS activation has been associated with some hallmarks of the DCM, including fibrosis and apoptosis [5,6]. RAAS blockers based on angiotensin-II receptor inhibition improved fibrosis and diastolic dysfunction in asymptomatic diabetic patients [1]. However, given the pleiotropic role of angiotensin-II [7] the downstream RAAS effector aldosterone may be considered as an alternative target. In this regard, aldosterone promotes angiotensin-II actions and fibrosis in the diabetic myocardium by up-regulation of pro-fibrotic and oxidative mediators [8]. Aldosterone exerts also apoptotic responses mainly by mitochondrial-dependent mechanisms [9], and these effects are worsened in hyperlipidemia and obesity [1,6]. Thus, eplerenone, a specific MR blocker, has demonstrated anti-fibrotic and anti-apoptotic properties in left ventricular hypertrophy, hypertension, and myocardial infarction [8,10]. Also, in controlled randomized clinical trials, eplerenone reduced mortality in patients with heart failure, independently of hypertension improvement and on top of angiotensin-II inhibition [11]. However, eplerenone actions on DCM and its related molecular mechanisms, particularly in steatosis and apoptosis, have not been elucidated.

## Methods

### Animal model

An obese non-hypertensive model of T2DM was used for this study (see Additional file 1). Zucker Diabetic Fatty (ZDF) rats lead to obesity and insulin resistance due to the inherited homozygous leptin receptor mutation (*fa*/*fa*) [12]. At the 14^th^ week, male ZDF rats were randomized and received eplerenone [25 mg/kg/day] or vehicle. N = 10, each group. Body weight and systolic blood pressure were monitored. After 16 weeks of treatment, blood and perfused hearts were isolated under anaesthesia. Plasma and renal parameters were measured in the Biochemistry Department of the Hospital. Hearts were rinsed, dried and weighted. Some ventricular slices were embedded in p-formaldehyde (to paraffin inclusion) or optimal-cutting-temperature (OCT) compound, for histology. Left ventricles were frozen in liquid-N_2_ for biochemical experiments. These investigations adhered to the Guide for the Care and Use of Laboratory Animals (NIH Publication No. 85–23, revised 1996) and the Ethics Committee of the hospital granted approval for these experiments.

### Cardiac structure and function

Cardiac echocardiography was performed under 1.5% isoflurane-O_2_ anaesthesia in all rats before (not shown) and after the treatment. Both M-mode and two-dimensional (2D) echocardiograms were obtained using a 12 MHz ultra-band sector transducer (Doppler). Images were obtained from the left and right parasternal window in a supine decubitus position. The following parameters were measured and calculated from M-mode tracing: left ventricular (LV) end-diastolic diameter (LVDD), LV end-systolic diameter (LVSD), ejection fraction (EF), deceleration time and the ratio of the early (E) to late (A) ventricular filling velocities. Wall thickness of four segments [anterior, inter-ventricular-septum (IVS), lateral, and posterior (LVPW) walls] was evaluated on short axis 2D images.

### Examination of cardiac fibrosis, steatosis, apoptosis and oxidative stress

Paraffin sections (4 μm) of all myocardia were fixed on slides and used for histology (see Additional file 1). Cardiac fibrosis was evaluated by Masson trichrome (Bio-Optica, Milan, Italy) staining. All forms of fibrosis (interstitial, perivascular and replacement fibrosis) were quantified together on ten fields of each myocardium using the Metamorph software. For neutral triglycerides and lipids determination, frozen OCT-sections were sliced (5 μm), immersed in propylene glycol and incubated in Oil red O (ORO) stain. Slides were transferred to propylene glycol and nuclear-counterstained with haematoxylin and mounted. Apoptosis was detected a TUNEL-based apoptosis detection Kit. The percentage of TUNEL-positive nuclei relative to total nuclei was determined in a blinded manner by counting 200-300 cells on ten randomly chosen fields per coverslip for each myocardium. Dihydroethidium (DHE; 5 μM, Invitrogen) was used to quantity cytosolic ROS production in paraffin-fixed myocardia. The average nuclear fluorescence intensity was measured in five fields of 50 cells by Metamorph. MitoSOX Red (5 μM, Invitrogen) was used to measure mitochondrial ROS production in myocardia. Paraffin sections were fixed on slides and incubated with MitoSOX Red (15 min at RT and darkness). Slides were stained 30′ with 4′,6-diamidino-2-phenylindole (DAPI), washed and mounted.

### Cultured cardiomyocytes

H9c2(2-1) is a permanent myoblast cell line derived from embryonic BD1X rat heart tissue (ATCC; USA). H9c2 were grown at 37°C in 5% CO_2_ in Dulbecco’s modified Eagle’s medium supplemented with 10% heat-inactivated foetal calf serum, 100 IE/ml penicillin, 100 μg/ml streptomycin, 2 mM L-glutamine and 5 mM D-glucose (Sigma). H9c2 differentiated from mononucleated myoblasts into myocytes upon overnight reduction of serum concentration before stimulation. Mouse C2C12 myoblasts (ATCC, USA) were kindly given by Dr. Konhilas (University of Arizona, USA) and maintained in DMEM supplemented with 10% foetal calf serum, 50 U/ml penicillin, and 50 μg/ml streptomycin. Before confluency, the medium was switched to the differentiation medium containing DMEM and 2% horse serum. After four additional days, the differentiated C2C12 cells fuse into myotubes. The hyperlipidemic or hyperglycemic conditions were mimicked by incubation with high concentrations of a common saturated FFA (Na^+^-palmitate, 16:0, 0.12-0.25 mM, Sigma) or glucose (D-glucose, 33 mM), respectively, for 12 h (protein expression) or 6 h (mRNA expression). Palmitate was previously conjugated with BSA in a 3:1 molar ratio as published elsewhere [13]. In control cells, BSA was added as described but in the absence of palmitate. Eplerenone (1 mM-1 μM) was added 1 h before stimulation.

### Glucose uptake

For glucose uptake evaluation, cardiomyocytes were grown under normal conditions and incubated for 3 hours with 100 μM 2-(N-(7-nitrobenz-2-oxa-1,3-diazol-4-yl)-amino-2-deoxyglucose (2-NBDG; Invitrogen) and HF or insulin, as a positive control. After discharging media and washing the cells, fluorescence was measured in the cytometer.

### ATP determination

Cellular ATP levels were quantified using a luciferase-based assay. Cardiomyocytes were exposed to HF (+/- eplerenone pre-treatment), after which, cells were rinsed with PBS and lysed with ATP-releasing buffer (100 mM KH_2_PO_4_, 2 mM EDTA, 1 mM dithiothreitol, and 1% Triton X-100 at pH 7.8). Ten μl of the lysate were taken for protein determination and another ten μl were used for ATP quantification using the ATP determination kit (Invitrogen), according to the manufacturer’s instructions.

### Detection of lipid-accumulation, apoptosis/survival and oxidation in cardiomyocytes

For cell steatosis quantification, after 12 h of stimulation cells were methanol-fixed and stained with ORO, as it was in myocardia. Lipid accumulation was semi-quantified by using Metamorph software on five fields of stimulated cells of at least three independent assays. Apoptosis was quantified by flow cytometry of cell DNA content (see Additional file 1). After stimulations, cells were harvested, permeabilized and DNA-stained with propidium iodide. The percentage of apoptotic cells is shown. Cells were also cultured in chamber slides, stimulated, fixed and nuclear-stained with DAPI. Condensed, piknotic and fragmented nuclei of apoptotic cells were identified by confocal microscopy (see Additional file 1). Cell survival was achieved with a MTT [3-(4,5-dimethylthiazol-2-yl)-2,5-diphenyltetrazolium bromide] Cell Growth Assay Kit, following manufacture’s instructions. MitoSOX Red was used to quantify mitochondrial ROS production. Cardiomyocytes were fixed on slides and incubated with MitoSOX Red, as it was in hearts. In addition, mitochondrial superoxide was evaluated by flow cytometry. Cells were grown in 10% FBS-DMEM without red phenol until sub-confluency. After overnight starvation, cells were stimulated, loaded with MitoSOX Red as it was in myocardia, and trypsin-detached. Cells were counted in the cytometer. Red fluorescence was measured at several intervals of time from confocal images. One mM H_2_O_2_ was used as control (data not shown).

### Lipid quantification

Portions of left ventricle myocardium or ~8×10^5^ stimulated cardiomyocytes were dissolved in were dissolved in 25 μL ethanol/mg or 100 μL ethanol, respectively. One glass bead (acid-washed, 2 mm, Sigma) was added to every tube and lipids were then extracted by vigorous shaking with a TissueLyser LT from Qiagen (Hilden, Germany) for 5 min, at 50 rpm. Tubes were further centrifuged at 15,400 g and 15°C for 20 min, and 80 μL from the supernatant were transferred to Ultra High Performance Liquid Chromatography-Mass (UHPLC-MS) spectrometry vials with insert. Quantitative evaluation of the proportion of lipids was performed by a spectrometer from Agilent Technologies (Santa Clara, CA, USA), equipped with a 1290 series LC system and a 6550 iFunnel QTOF MS detector. One μL from each sample was injected (three times) onto the column, a Zorbax Eclipse Plus C8, 2.1 × 150 mm; 1.8 μm (Agilent Technologies) kept at 80°C. Compounds were eluted with an 8 min linear gradient for the mobile phase at 0.6 mL/min, from 50% ammonium formate 10 mM (pH 6.5) and 50% Methanol to 100% Methanol. Conditions, reagents, as well as the procedure for data processing in order to obtain a list of abundances of all the lipids of interest are provided in Additional file 1.

### Western blot and ELISA

A piece (50 mg) of homogenized ventricle (Bullet Blender, Cultek) or cell extract were dissolved in cold lysis buffer (see Additional file 1). Equal amounts of proteins (20-30 μg) were separated on polyacrylamide gels, transferred to membranes and probed with primary antibodies. Secondary HRP-linked antibodies (GE Healthcare) were used for chemo-luminescence development. A representative gel of all rats or at least three independent experiments of cultured cells with the semi-quantification scores (n-fold vs. GAPDH) are shown. Following manufacture’s guidelines, rat endogenous aldosterone and glucocorticoids ELISA kits (antibodies-online.com) were used for aldosterone and glucocorticoids detection in cultured media, respectively. Plasma insulin was detected using a rat insulin ELISA kit (Mercodia, Sweden).

### Quantitative-PCR (QPCR)

Total RNA was extracted from homogenized ventricle (50 mg) or cultured cardiomyocytes by dissolving in Trizol reagent (Invitrogen). Equal amounts of RNA (1 μg) were reverse-transcripted to obtain the cDNA for multiplex QPCR with specific probes (see Additional file 1). We show a quantification (n-fold vs. 18 s) of at least two QPCRs of all rats or three independent cultured cardiomyocytes experiments.

### Statistical analysis

Data are expressed as mean±standard deviation. Multiple comparisons were performed by non-parametric Kruskal-Wallis test followed by a Mann-Whitney test. A two-tailed p < 0.05 was considered significant.

## Results

### Reduction of hyperlipidemia by eplerenone in obese/T2DM rats

**Figure 1:**
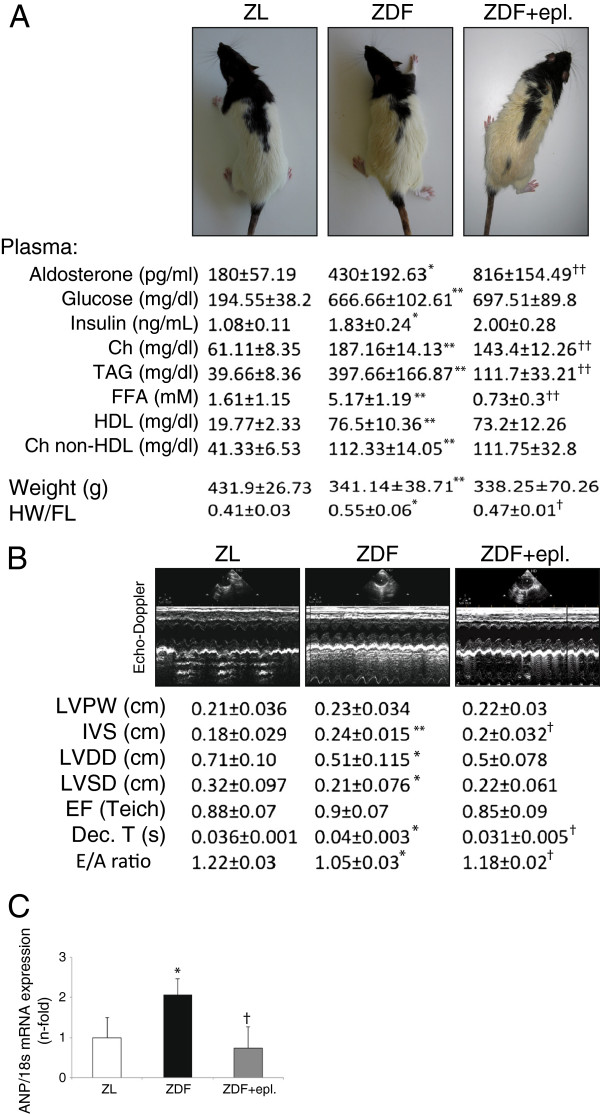
**Eplerenone improved hyperlipidemia and cardiac hypertrophy in ZDF rats.** After sixteen weeks of treatment, **(A)** physical and plasmatic parameters, and **(B)** cardiac structure and function in ZL, ZDF and ZDF-treated rats (n = 10, each group). Representative photographs of rats and Echo-Doppler images for each group are also shown. **(C)** Pro-hypertrophic ANP expression in the hearts. Ch, cholesterol; TAG, triglycerides; FFA, free fatty-acid; HDL, high-density lipoproteins; HW, heart weight; FL, femur length. LVPW, left-ventricular posterior wall and IVS, inter-ventricular septum (IVS) thicknesses; LVDD, left ventricular diastolic and LVSD, left ventricular systolic diameters; EF, ejection fraction and Dec. T, deceleration time. *p < 0.05 and **p < 0.01 vs. ZL. †p < 0.05 and ††p < 0.01 vs. ZDF rats.

After 30 weeks, ZDF rats exhibited high plasma levels of aldosterone, glucose, insulin and lipid profile (Figure 1A). Interestingly, 16 weeks of eplerenone substantially triggered plasma aldosterone and reduced plasma lipids. No significant changes in systolic blood pressure (135.0 ± 8.1 vs. 139.8 ± 15.2 mm Hg for ZL and ZDF rats, respectively) were observed. Markers of severe renal (urea, blood urea nitrogen, creatin and albumin) and liver (aspartyl and alanine aminotransferases) injury remained within the normal ranges in all groups (not shown). Of note, since mineralocorticoid antagonists may induce hyperkalemia [14], we monitored plasma potassium concentrations. Conveniently, at this dose/time of eplerenone, potassium was kept within the non-toxic levels in all rats (4.4 ± 0.58 mEq/l).

### Attenuation of cardiac hypertrophy and diastolic dysfunction by eplerenone in ZDF rats

As previously documented [15], at this stage of the disease ZDF rats exhibited weight loss. However, a significant elevation of the heart weight/femur length (HW/FL) ratio was observed (Figure 1A). Moreover, by Echo-Doppler (Figure 1B), ZDF hearts exhibited an increase of the inter-ventricular septum (IVS) thickness, and a reduction of left ventricular diastolic (LVDD) and systolic (LVSD) diameters, probably related to IVS hypertrophy. In this sense, the mRNA expression of atrial natriuretic peptide (ANP), a molecular marker of hypertrophy, was increased in the left ventricle of ZDF (Figure 1C). Brain natriuretic peptide (BNP) was, however, not significantly modified in the rats (not shown). In addition, ZDF showed a prolongation of the deceleration time and decreased E/A ratio, suggesting diastolic dysfunction. However, the ejection fraction (EF) was unchanged. Interestingly, the altered HW/FL, IVS, ANP expression, deceleration time and E/A ratio were attenuated by eplerenone administration.

### Eplerenone ameliorated fibrosis and apoptosis in the ZDF myocardium

**Figure 2:**
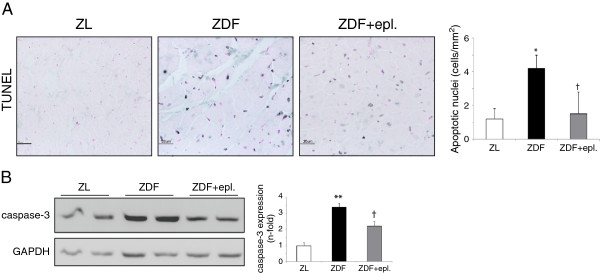
**Eplerenone attenuated apoptosis in the ZDF myocardium. (A)** By TUNEL, detection of apoptotic nuclei (dark blue) in the hearts. **(B)** Activation of caspase-3 (cleavage isoform; ~17 kDa) in ZDF and ZDF-treated rats. N = 10, each group. *p < 0.05 and **p < 0.01 vs. ZL. †p < 0.05 vs. ZDF rats.

Left ventricular myocardium in the ZL rats exhibited a normal architecture with regular interstitial space. However, abnormal myocardial architecture (cardiomyocyte hypertrophy and disarray, and enlarged interstitial space) was observed in the ZDF group (Additional file 2: Figure S1A). Confirming previous data in experimental [16] and human [17] diabetes, the deposition of interstitial and mainly perivascular extra-cellular matrix (ECM), and the increase of pro-fibrotic factors such as type-IV collagen and fibronectin, were attenuated by eplerenone (Additional file 2: Figure S1A, B). More interestingly, since both hypertrophy (Figure 1A-C) and fibrosis (Additional file 2: Figure S1A, B) may contribute to myocardial remodelling in response to a loss of cells [6,18], we focused on the apoptotic response of ZDF hearts and their treatment with eplerenone. By TUNEL (Figure 2A), we observed an increase of apoptotic-positive nuclei in ZDF (4.1 vs. 1.2 cells/mm^2^ ZL), which was reduced by eplerenone. Moreover, ZDF showed an activation of pro-apoptotic caspase-3, and eplerenone mitigated this response (Figure 2B).

### Eplerenone decreased apoptosis in high fatty acid-stimulated cardiomyocytes

**Figure 3:**
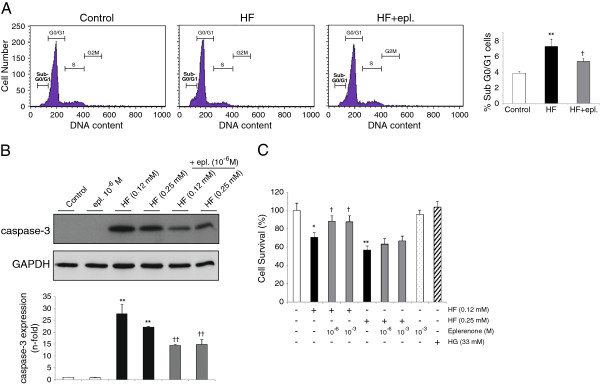
**Eplerenone reduced apoptosis in HF-stimulated cardiomyocytes. (A)** By flow cytometry, detection of apoptotic cardiomyocytes (sub G0/G1 phase) after HF (0.12 mM) and HF+eplerenone (10^-6^ M). **(B)** Caspase-3 activation. **(C)** Cell viability assayed by MTT in cardiomyocytes stimulated with HF and/or eplerenone. *p < 0.05 and **p < 0.01 vs. related control. †p < 0.05 and ††p < 0.01 vs. HF.

To elude the confounding effects of reduced hyperlipidemia on the eplerenone-treated ZDF myocardium, we tested the apoptosis response in eplerenone-pretreated cultured cardiomyocytes exposed to high concentrations of a saturated fatty acid (HF) and/or glucose (HG). In our conditions, HF, but not HG, significantly increased the number of apoptotic cardiomyocytes as early as 14 h incubation and at 0.12 mM (Additional file 2: Figure S1C), similarly to a lethal cytokine (30 U/ml IFNγ; not shown). In addition, caspase-3 activation and subsequent nuclear pyknosis and cell loss were detected mainly in HF-incubated cardiomyocytes (Additional file 2: Figure S1D). HF and HG co-incubation did not significantly alter the magnitude of HF-induced apoptosis. In addition, HF induced the release of aldosterone from cardiomyocytes to the cultured media (Additional file 3: Figure S2), and thus, eplerenone may block aldosterone-associated actions on cultured cardiomyocytes. In fact, eplerenone (1 μM) reduced HF-induced cell apoptosis and caspase-3 activation (Figure 3A, B). These data were confirmed by MTT survival assays. Incubation with HF significantly decreased the cardiomyocytes viability (black bars) and this effect was mitigated by eplerenone (only for 0.12 mM; Figure 3C). HG and eplerenone alone did not modify the survival rates.

### Eplerenone reduced myocardial steatosis in ZDF hearts

**Figure 4:**
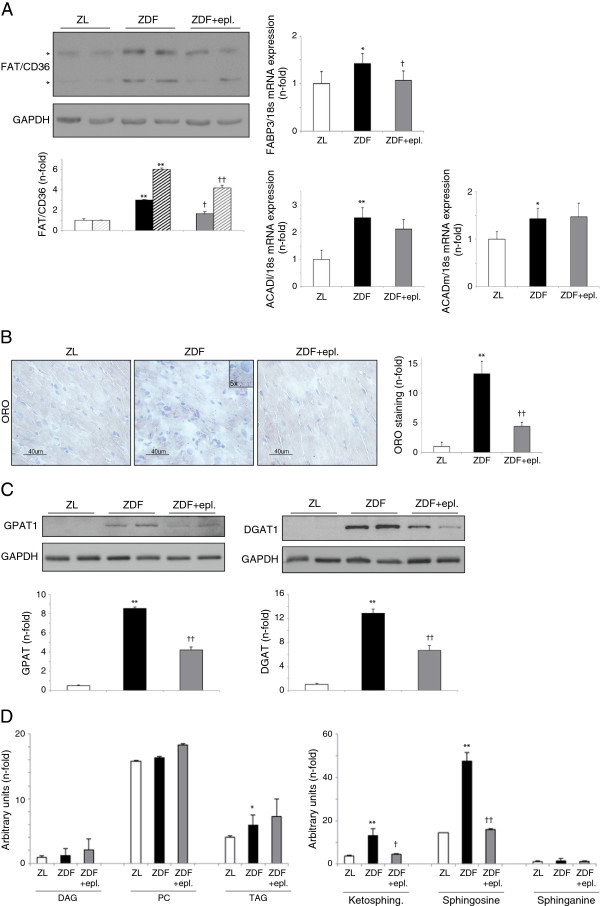
**Eplerenone decreased lipid steatosis and ceramides formation in ZDF hearts. (A)** Left, FAT/CD36 isoforms (~90 kDa and ~53 kDa; solid bars and broken bars in the quantification graph, respectively) expression in ZL, ZDF and ZDF+eplerenone hearts. Right, FABP3, ACADl and ACADm mRNA expression. **(B)** Detection of intra-cellular lipid deposition (steatosis) in the rats by ORO staining. A detailed 5x magnification of the lipid droplets is shown in ZDF. **(C)** Protein levels of GPAT1 (~90 kDa) and DGAT1 (~52 kDa) in rat myocardia. **(D)** By UHPLC-MS, DAG, phosphatidyl-choline (PC), TAG, and ketosphingosine, sphingosine and sphinganine levels. *p < 0.05 and **p < 0.01 vs. ZL. †p < 0.05 and ††p < 0.01 vs. ZDF rats.

Apoptosis in obese/T2DM hearts may result from an excessive uptake and accumulation of FFA [3,18]. The main cardiac FFA protein-transporter is FAT/CD36, which traffic from intracellular stores (endosomes) to the sarcolemma to facilitate FFA uptake [19]. Indeed, FAT/CD36 isoforms, likely corresponding to the non-glycosylated (lower) and glycosylated/phosphorylated (upper) proteins [20], were increased in the ZDF myocardium (Figure 4A, left). Next, FFA can deliver to different organelle, primarily to mitochondria for energy consecution. In this regard, ZDF showed elevated mRNA expression of a FFA-cytosolic transporter, the FFA-binding protein-3 (FABP3; Figure 4A, right) and two main β-oxidation enzymes, ACADl and ACADm (large chain- and medium chain- acyl-CoA dehydrogenases, respectively) (Figure 4A, right). However, as a consequence of high FFA-uptake (or uncoupled FFA-uptake and oxidation), FFA can be stored by re-esterification as TAG and phospholipids in the cytosol of obese/T2DM cardiomyocytes [21]. In this sense, we identified numerous cytosolic lipid droplets (Figure 4B) in ZDF myocardia, typically from myocardial steatosis. Also, ZDF up-regulated two rate-limiting enzymes of lipid re-esterification, GPAT1 (glycerol-3-phosphate acyltransferase-1) and DGAT1 (DAG acyltransferase-1) (Figure 4C), and increased TAG content (Figure 4D, left). Then, if this accumulation persists overt time, lipids may diverge to lipotoxic metabolites such as ceramides [22,23]. In this regard, ZDF hearts accumulated sphingolipids precursors of ceramides such as ketosphingosine and sphingosine (Figure 4D, right). Interestingly, eplerenone lessened the expression/translocation of FAT/CD36 and FABP3, myocardial steatosis, GPAT1/DGAT1, and ceramides intermediates (Figure 4A-D).

### Eplerenone decrease lipid metabolism and accumulation in HF-stimulated cardiomyocytes

**Figure 5:**
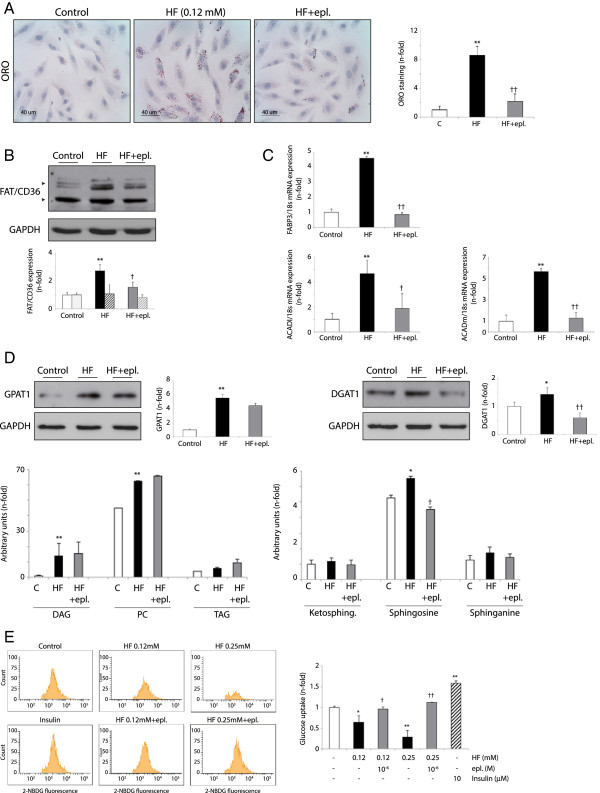
**Eplerenone reduced lipid metabolism and ceramides formation in cultured cardiomyocytes. (A)** Lipid accumulation in control, HF- and HF+eplerenone-treated H9c2. **(B)** FAT/CD36 protein content in C2C12 myotubes (~90 kDa and ~53 kDa; solid bars and broken bars in the quantification graph, respectively). **(C)** FABP3, ACADl and ACADm transcript levels. **(D)** Top, GPAT1 and DGAT1 protein expression. Bottom, DAG, PC, TAG, and ketosphingosine, sphingosine and sphinganine levels by UHPLC-MS. **(E) Eplerenone improved glucose assimilation in HF-incubated C2C12**. Eplerenone was pre-administrated in some HF- or insulin-stimulated cells, and 2-NBDG was evaluated. *p < 0.05 and **p < 0.01 vs. related control. †p < 0.05 and ††p < 0.01 vs. related HF.

Cardiac steatosis was confirmed in HF-incubated cardiomyocytes by Oil Red-O staining (Figure 5A). However, previous data had suggested that H9c2 cells might not express FAT/CD36 receptor [24]. Thus, we used another cell line of myocytes with demonstrated FAT/CD36 expression and activated lipid metabolism [25]. C2C12 cells increased also lipid droplets (not shown), and up-regulated glycosylated/phosphorylated FAT/CD36 isoforms after HF incubation, possibly corresponding to its sarcolemma translocation (Figure 5B). Downstream, FABP3 and ACADl/ACADm transcripts were augmented by HF (Figure 5C). GPAT1/DGAT1 (Figure 5D, top), DAG, phospholipids (phosphatidyl-choline) and sphingosine were also increased (Figure 5D, bottom). However, in agreement with ZDF-treated rats, eplerenone mitigated cytosolic lipid accumulation, FAT/CD36 and FABP3 after HF (Figure 5A-C). Eplerenone reduced also DGAT1 and sphingosine (Figure 5D). These data suggest a direct effect of eplerenone on lipid uptake and storage at the myocardium.

By other hand, unsurprisingly, HG did not induce steatosis in cardiomyocytes (not shown). However, the stimulated lipid storage and metabolism could affect glucose utilization [21]. In this sense, as soon as 3 h of HF-incubation, C2C12 exhibited a significant decrease of glucose uptake that was reverted by eplerenone (Figure 5E).

### Eplerenone improved DCM-associated mitochondrial stress but not mitochondrial regulators

**Figure 6:**
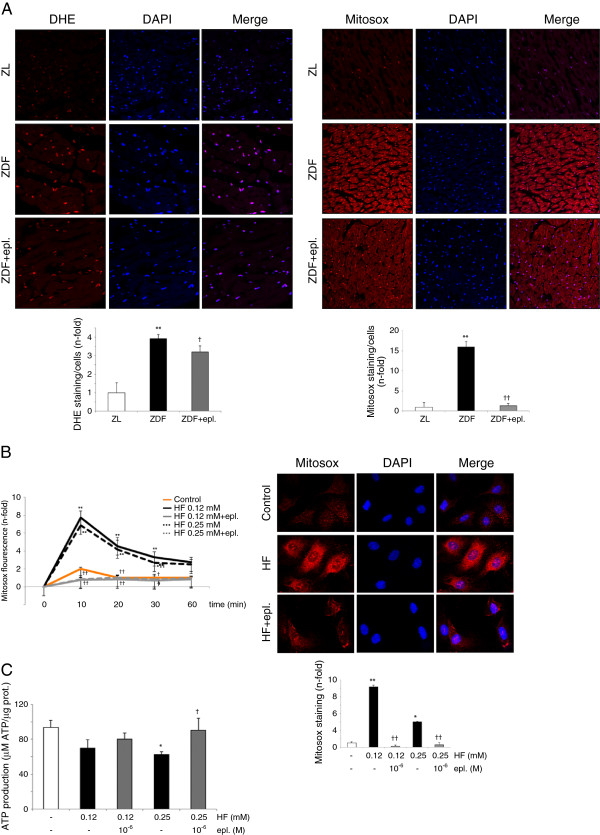
**Anti-oxidant effects of eplerenone in ZDF hearts and HF-stimulated cardiomyocytes. (A)** DHE and Mitosox staining (red) in ZDF and ZDF-treated myocardia. Nuclear labelling by DAPI (blue) is also shown. N = 6, each group. **(B)** By flow cytometry (left), mitosox staining in 0-60 min HF and HF+eplerenone (10^-6^ M) incubated cardiomyocytes. By immunofluorescence (right), mitosox staining after 10 min of HF stimulation (0.12 mM). **(C)** ATP production in 12 h HF-stimulated cardiomyocytes. *p < 0.05 and **p < 0.01 vs. control. †p < 0.05 and ††p < 0.01 vs. related ZDF or HF.

Apoptosis in the obese/T2DM heart has been also related to an overproduction of reactive oxygen species (ROS), which damages cell mitochondria and induce apoptosis [3]. In our model, ZDF showed an increase of cytosolic (Figure 6A, left) and mainly mitochondrial (Figure 6A, right) superoxide production, but eplerenone attenuated these levels. These data were confirmed in cultured cardiomyocytes. By flow cytometry (Figure 6B, left) and immunofluorescence (Figure 6B, right), HF induced mitochondrial-ROS formation as early as 10 min of stimulation, and eplerenone prevented this effect. Consistently, mitochondrial ATP production was decreased in HF-incubated cardiomyocytes (0.25 mM), and restored after eplerenone treatment (Figure 6C).

**Figure 7:**
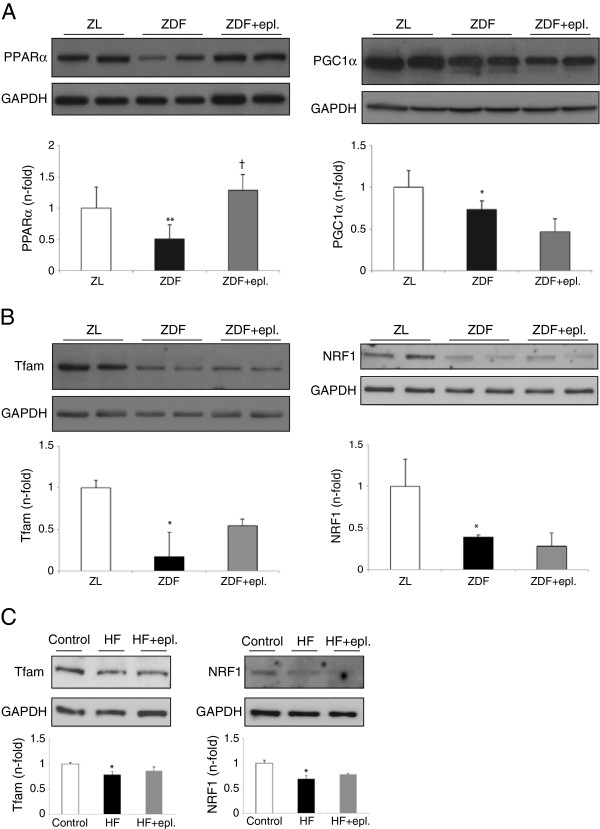
**Mitochondrial-related factors in ZDF myocardia and HF-incubated cardiomyocytes. (A)** PPARα (~50 kDa), PGC1α (~120 kDa), and **(B)** Tfam (~25 kDa) and NRF1 (~55 kDa) levels in the ZDF model. **(C)** Tfam and NRF1 protein content in 12 h HF-stimulated cardiomyocytes. *p < 0.05 and **p < 0.01 vs. control. †p < 0.05 vs. related ZDF or HF.

More interestingly, the levels of key regulators of mitochondrial function such as the complex peroxisome proliferator activated receptor-α (PPARα)/PPARγ coactivator-1α (PGC1α), and related transcription factors involved in mitochondrial biogenesis and oxidative metabolism, such as the mitochondrial transcription factor-A (Tfam) and the nuclear respiratory factor-1 (NRF1) [26], were significantly reduced in ZDF hearts (Figure 7A-B). However, only PPARα ωασ restored by eplerenone. Interestingly, HF (0.25 mM) did not alter PPARα/PGC1a (not shown), but reduced the content of Tfam and NRF1 in H9c2. Again, eplerenone could not prevent these responses (Figure 7C).

## Discussion

Elevated content of lipids in cardiac muscle, and following increase of apoptosis and ECM deposition are distinctive of human and experimental DCM. These abnormalities may generate cardiac hypertrophy and dysfunction, eventually leading to congestive heart failure [1,21]. Chronic obese/T2DM rats exhibited cardiac steatosis, apoptosis, fibrosis and hypertrophy, and diastolic (but not systolic) dysfunction. In DCM, diastolic dysfunction is not necessary accompanied with a reduction of EF [12,27], or it occurs earlier than systolic alteration [28]. Nevertheless, a valid treatment for DCM patients is needed. As we confirmed here, the blockade of MR has emerged as an effective anti-fibrotic therapy in DCM [5,29]. However, we also claim it might induce anti-steatosis and anti-apoptosis actions, contributing to the recovery of diastolic function [2].

**Figure 8:**
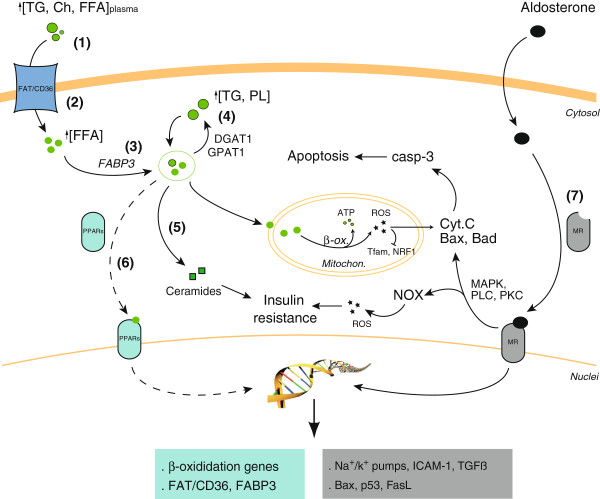
**Potential protective effects of eplerenone in chronic obese/T2DM hearts.** In cardiomyocytes, plasma lipids can be uptake as FFA to undergo mitochondrial β-oxidation for energy supply. An overload of FFA causes increased oxidation and ROS, mitochondrial damage and apoptosis. FFA may also accumulate as TAG and phospholipids (PL) in lipid droplets, or deviate to ceramides. However, PPARα activation may be ameliorated in these cells (dotted lines). In addition, released aldosterone may bind cytosolic MR and induce non-genomic and genomic effects on pro-oxidative, apoptotic and fibrotic factors. Interestingly, eplerenone may mitigate hyperlipidemia (1), FFA-assimilation (2), delivering (3) and lipid-metabolites formation (4) (5), induce PPARα activation (6) and reduce MR-associated actions (i.e., insulin resistance) (7). NOX, NADPH-oxidase; MAPK, MAP-Kinases; PKC, protein kinase-C; PLC, phospholipase-C.

Long-term hyperglycemia promotes negative effects on the heart, affecting the cardiac expression of lipid-, glucose- and ketone bodies-metabolic, signalling and stress-response genes [30,31]. Chronic hyperglycemia is related with cardiac fibrosis, coronary disease and increased risk of heart attack, and it has been also associated with higher levels of toxic glycolytic intermediates and troponin-T, a blood marker for heart damage [32]. However, recent clinical trials have revealed non-significant advances of intensive glycemic control on the mortality of DCM patients with cardiovascular events [33], suggesting that other events such as lipotoxicity may be targeted for new therapies. In this sense, eplerenone reduced hyperlipidemia without altering hyperglycemia in ZDF rats (Figure 8). Similar data were found in obese/T2DM mice [34], and individuals with essential hypertension [35]. Thus, the anti-hyperlipidemic actions of eplerenone could be independent of changes in glycemia or blood pressure [36], and possibly due in part to an improvement of insulin resistance. After MR blockade, experimental and clinical studies have demonstrated an increase of the insulin response by reduction of redox-sensitive and -insensitive serine kinases, and consequent activation of IRS-1 signaling. Also, the MR blockade increased both insulin and glucose receptor genes, leading to improved glucose uptake [37][38]. Here we also showed a restoration of IRS-1 levels (Additional file 3: Figure S2B) and glucose uptake in eplerenone-treated ZDF or HF-exposed cardiomyocytes, respectively. Nevertheless, the role of eplerenone on lipid intestinal absorption and release from adipose tissue is not clarified. In this sense, eplerenone prevented adiponectin reduction and leptin elevation of adipose tissue in obese/T2DM mice [34].

The attenuated plasma lipid availability could bring about a reduction of lipid deposition within the heart. However, we found potential direct anti-steatotic effects in DCM (Figure 8). The expression/sarcolemma relocation of FAT/CD36 (and FABP3) was ameliorated with eplerenone in ZDF hearts, but also in HF-incubated cardiomyocytes. Interestingly, FAT/CD36, FABP3, and several β-oxidation enzymes, are transcriptional targets of PPARα [39], however, PPARα was elevated after eplerenone in ZDF hearts. The metabolic changes associated to the activity of PPARα may depend on the stage of obesity and diabetes [40,41]. Chronic exposure to elevated FFA reduced PPARα in cardiomyocytes, and this effect was proposed to further decrease cardiac function and increase intracellular fat stores [21]. Also, the PPAR-response elements were not identified on FAT/CD36 gene [42], and PPARα may need specific coactivators such as PGC1α to mediate these actions [43]. Of interest, PGC1α was also not stimulated in ZDF. Next, we observed an increase of cytosolic lipid droplets and activation of lipid re-esterification in ZDF myocardia and HF-incubated cardiomyocytes (Figure 8). This effect may be related to the rise of TAG, and DAG and PC, respectively. Moreover, we detected an accumulation of sphingosine in both ZDF myocardia and HF-exposed cells. The increased lipogenic capacity [21,44], and overall, the cardiac deposition of ceramides could promote insulin resistance, lipoapoptosis and dysfunction in the ZDF heart [22,23]. However, eplerenone reduced lipid droplets, lipid re-esterification enzymes and ceramides formation, likely contributing to the improved diastolic dysfunction (Figure 8).

Finally, a lipid overload together with a reduction in glucose assimilation may also result in non-neutralized mitochondrial ROS production and apoptosis in DCM (our data and [41]). In ZDF myocardia and HF-exposed cardiomyocytes we have described an increase of cytosolic and mainly mitochondrial ROS, consequent ATP deficiency, and apoptosis. These levels correlated with the reduced expression of PPARα/PGC1α complex and linked transcription factors (Tfam and NRF1). Interestingly, eplerenone attenuated ROS levels, ATP deficiency and apoptosis, without altering these mitochondrial regulators, and thus, possibly due at least in part to the lipotoxicity lessening. Eplerenone decreased also β-oxidation in HF-incubated cells, as a source of ROS. Additionally, previous works also demonstrated protective properties of eplerenone in hyperosmotic cardiomyocytes [45], and some pro-apoptotic and pro-inflammatory/oxidative factors may be involved [46,47]. Altogether, by MR blockade and steatosis reduction, eplerenone could decrease aldosterone- and FFA-associated pro-oxidative, apoptotic and fibrotic actions [34] (Figure 8). Further investigations focusing on these particular mechanisms will add new insights to the knowledge of eplerenone protection in DCM. However, long-term eplerenone administration could lead to hyperkalemia, resulting in depolarization of the membrane potentials of cardiac cells and fatal arrhythmias [14]. Also eplerenone may induce off-target effects through the aldosterone competitive antagonism of the androgen receptor, affecting hormone secretion and function [48].

### Study limitations

Glucocorticoids were detected in cultured media (Additional file 3: Figure S2A). In particular, plasma cortisol was ~30% higher in ZDF than in ZL rats. Since glucocorticoids can also bind to MR, we cannot exclude their potential effects in hearts and cardiomyocytes. In addition, the quantification of food intake could add important information since the decreased plasma FFA/TAG levels after eplerenone may be caused by differences in food consumption. Nevertheless, previous data in rat demonstrated no variation in food and water consumption after eplerenone administration [49].

## Conclusions

Intracellular accumulation of lipids in the experimental obese/T2DM heart appears to play an important role in the pathogenesis of DCM. However, eplerenone decreased hyperlipidemia, myocardial FFA-uptake and steatosis, insulin resistance, and ceramides and ROS accumulation, which all may contribute to the improvement of energy consecution, cardiac remodelling and function. Even in the presence of high glucose concentration, our work supports the importance of controlling myocardial lipotoxicity for preventing the development of DCM, and eplerenone could attend as a valid therapy.

## Competing interests

The authors declare that they have no competing interests.

## Authors’ contributions

ER and MK-M carried out the most of the molecular studies. SA-C performed the animal model and AC-V measured the cardiac structure and function. BP participated in the histological approaches. FJR, AF and CB performed lipid quantification. JE, JT and OL designed the study and performed the statistical analysis. OL coordinated and wrote the work. All authors read and approved the final manuscript.

## Supplementary Material

Additional file 1: Supplemental Material Details of the ZDF model and used techniques. Click here for file

Additional file 2: Figure S1 Eplerenone attenuated fibrosis and ECM proteins in the ZDF myocardium. (A) By Masson, detection of ECM deposition (blue-green) in ZDF and ZDF+eplerenone hearts (top). (B) Type-IV collagen and fibronectin mRNA expression in the rats. N = 6, each group. (C) HF induced apoptosis in cultured cardiomyocytes. Cardiomyocytes were stimulated with HF (0.12-0.5 mM) or glucose (HG, 25-33 mM) for 3-24 h, and apoptosis was quantified by flow cytometry. The percentage of apoptotic cells (sub G0/G1 cell cycle phase) is represented. (D) Caspase-3 activation after 3-14 h of HF and/or HG incubation, and nuclei piknosis (detailed in a bright field) and cell loss in 14 h-stimulated cardiomyocytes. *p < 0.05 and **p < 0.01 vs. related control. †p < 0.05 and ††p < 0.01 vs. ZDF rats. Click here for file

Additional file 3: Figure S2 (A) Aldosterone and glucocorticoids release from cardiomyocytes. Aldosterone and glucocorticoids (GC) were measured in cultured media after 12 h HF and HF+eplerenone incubations. (B) Cardiac IRS-1 expression. Total IRS-1 levels (~130 kDa) were detected in rat myocardia. *p < 0.05 vs. related control. ††p < 0.01 vs. ZDF rats. Click here for file
