# Supplementary material for: Eplerenone attenuated cardiac steatosis, apoptosis and diastolic dysfunction in experimental type-II diabetes
Source: Cardiovasc Diabetol. 2013 Nov 21;12:172. doi: 10.1186/1475-2840-12-172 (PMC4222723; doi:10.1186/1475-2840-12-172)
Supplement: Additional file 1: Supplemental Material — Details of the ZDF model and used techniques. [file 1475-2840-12-172-S1.docx]

**Additional file**

**Methods:**

**Animal model**

Male ZDF rats were purchased from Charles Rivers (France) and were kept on an artificial 12-hour light-dark cycle (7 a.m.-7 p.m.) at 25°C. Under fatty diet intake (Purina #5008; 26.8% protein, 16.7% fat and 56.4% carbohydrates), ZDF rats, but not their lean littermates (Zucker Lean), developed obesity and type-II diabetes. Rats received tap water *ad libitum*. After full onset of type-II diabetes (at the 14^th^ week), ZDF rats were randomized and received eplerenone [Pfizer; 25 mg/kg/day in drinking water, by oral gavage (10 a.m.)] or vehicle. Systolic blood pressure was monitored by tail-cuff method. After 16 weeks of treatment, plasma (collected from cava vein) and PBS-perfused hearts were isolated under isoflurane (1.5% in O_2_) anaesthesia (3-7 p.m.). Plasma lipid profile, aldosterone, cortisol, glucose, hepatic enzymes, ions and renal parameters were measured in the Biochemistry Department of the Hospital.

**Examination of cardiac steatosis, apoptosis and oxidative stress**

Frozen OCT-sections were sliced with the cryostat, immersed in 100% propylene glycol (2’) and incubated (60°C, 1h) with constant stirring in filtered and preheated 0.7% Oil red O (ORO) stain (Sigma-Aldrich; USA) for cardiac steatosis evaluation. Slides were transferred to 80% propylene glycol, washed in distilled water, nuclear-counterstained with haematoxylin and mounted in glycerol jelly. *In situ* DNA fragmentation was identified by incorporating labeled nucleotides onto the free 3'-OH ends of DNA fragments and using a terminal deoxynucleotidyl transferase dUTP nick end labeling (TUNEL)-based CardioTACS In Situ Apoptosis Detection Kit (R&D systems; Minneapolis, USA). DNA was visualized by binding streptavidin-horseradish peroxidase followed by reaction with TACS Blue Label™, which generated a dark blue precipitate. DHE was used myocardial cytosolic ROS quantification. DHE is a cell-permeable fluorescent dye that is oxidized by cytosolic superoxide to fluorescent ethidium bromide, which is a nucleic acid stain trapped by intercalation with DNA.

**Detection of apoptosis and survival in cardiomyocytes**

After stimulations, H9c2 were harvested, permeabilized in PBS-0.05% NP-40 and 10 μg/ml RNAse-A (3h, 4°C), and DNA-stained with 100 μg/ml propidium iodide for apoptosis measurement by flow cytometry (FACSCalibur Analyzer, BD). Lower DNA content (hypodiploid cells; Sub-G0/G1 phase) due to nuclear fragmentation characterized apoptotic cells. The percentage of apoptotic cells with decreased DNA content is shown. Cells were also cultured in chamber slides (Nunc; Naperville, IL), stimulated, fixed with methanol:acetone (1:1) and nuclear-stained with DAPI. Condensed, piknotic and fragmented nuclei of apoptotic cells were identified by a laser scanning confocal microscope (Leica). MTT [3-(4,5-dimethylthiazol-2-yl)-2,5-diphenyl tetrazolium bromide; Sigma] is cleaved by living cells to yield a dark blue formazan product detected by a colorimetric assay (Abs_540nm_). Bioreduction of MTT involves endoplasmic reticulum, cytosolic and plasma membranes reductases. MTT was added to cells at a concentration of 0.5 mg/ml for 3h at 37°C, and the number of cells in the bottom of each plate exhibiting a positive blue granular reaction product was quantified.

**Lipid quantification: Data processing and reagents**

The resulting data files were processed with MassHunter Qualitative Analysis B.05.00 in order to clean of background noises and unrelated ions by the Molecular Feature Extraction (MFE) tool. Data pre-treatment including alignment and filtering was performed in MassProfiler Professional (B.12.01, Agilent Technologies). The result was a matrix with all the compounds in the samples sorted by their characteristic retention time and neutral mass, and the abundance of each compound for each sample. The list of exact masses was exported to public databases (METLIN – <http://metlin.scripps.edu/>; LipidMaps - <http://www.lipidmaps.org/>; and KEGG - <http://www.genome.jp/kegg/>), all accessed through our own developed engine, CEU Mass mediator (<http://biolab.uspceu.com/mediator/>) and the features for every lipid were identified by the exact mass (less than 5 ppm error).

MS detection conditions:

- Fragmentor: 175 V
- Skimmer: 65 V
- Gas Temp.: 250 °C
- Drying Gas 12 ml/min
- Nebulizer: 52 psig
- Sheath Gas Temp: 370 °C
- Sheath Gas Flow: 11 ml/min
- Capillary voltage: 3000 V
- Nozzle Voltage: 1000 V
- Reference masses: 121.0509 m/z (C5H4N4) and 922.0098 m/z (C18H18O6N3P3F24)

Ethanol (HPLC-grade) and ammonia (Reagent-grade) were purchased from Panreac Quimica (Castellar de Vallés, Barcelona, Spain), formic acid (98%) and LC-MS grade methanol were purchased from Fluka Analytical (Steinheim, Germany).

**Western Blot**

Lysis buffer was: 50 mM Tris-HCl pH 7.5, 1 mM EDTA, 2% SDS + 1/250 mammalian protease inhibitors. Protein content of blood-free heart tissue homogenates and cultured cardiomyocytes were detected by primary antibodies anti-FAT/CD36, -IRS-1, -SPTLC2, -DGAT1, -GPAT1, -PPARα -PGC1α, -Tfam, -NRF1 (Sigma) and -caspase-3 (Cell Signalling). Anti-GAPDH (Millipore, MA, USA) was used as loading control.

**QPCR conditions**

#### A mixture of QPCR was prepared as it follows: 33 ng of cDNA, 0.25 μl of gene expression assays [0.125 μl ANP (Rn00561661_m1), BNP (Rn00580641_m1), type-IV collagen (Rn01401018_m1), fibronectin (Rn00569575_m1), FAT/CD36 (Rn00580728_m1), FABP3 (Rn00577366_m1), ACADl (Rn00563121_m1), ACADm (Rn00566390_m1) Fam-fluorophore + 0.125 μl housekeeping gene eukaryotic ribosomic 18s Vic-fluorophore (4310893E)], 5 μl premix buffer (polymerase and salts) and RNAase free water (TaqMan; Applied Biosystems). Amplification conditions were: 2’ at 50°C, 10’’ at 95°C and 40 cycles of 15’’ at 95°C and 1’ at 60°C. All samples were prepared in triplicate to obtain their threshold cycle (Ct). If deviation for each triplicate were higher than 0.3 cycles, Ct was not considered. The relative expression for each gene was achieved following the model R=2^-ΔΔCt^.
