# Supplementary figures and images for: Eplerenone attenuated cardiac steatosis, apoptosis and diastolic dysfunction in experimental type-II diabetes
Source: Cardiovasc Diabetol. 2013 Nov 21;12:172. doi: 10.1186/1475-2840-12-172 (PMC4222723; doi:10.1186/1475-2840-12-172)

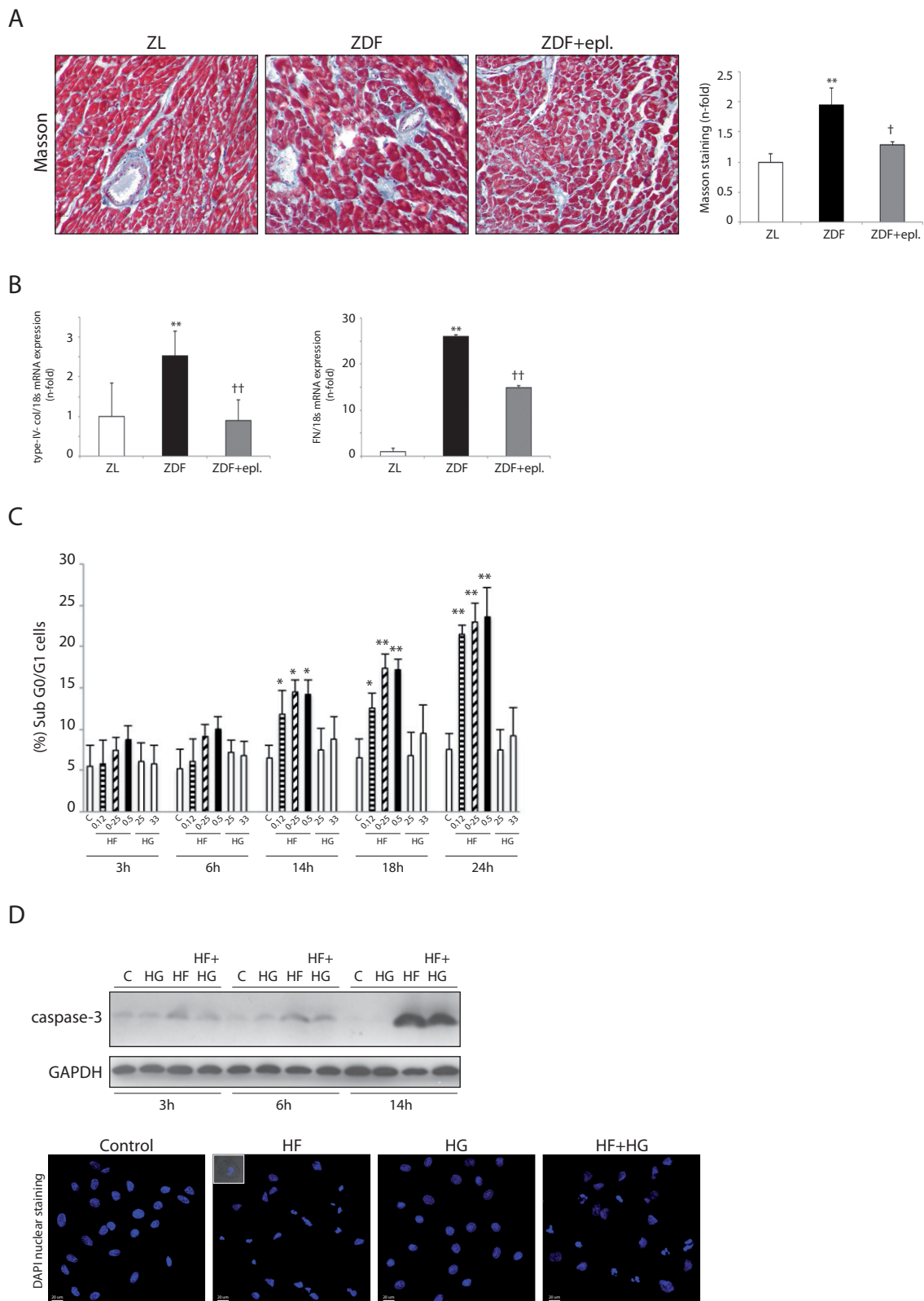

Suppl. Figure 1.

Supplement: Additional file 2: Figure S1 — Eplerenone attenuated fibrosis and ECM proteins in the ZDF myocardium. (A) By Masson, detection of ECM deposition (blue-green) in ZDF and ZDF+eplerenone hearts (top). (B) Type-IV collagen and fibronectin mRNA expression in the rats. N = 6, each group. (C) HF induced apoptosis in cultured cardiomyocytes. Cardiomyocytes were stimulated with HF (0.12-0.5 mM) or glucose (HG, 25-33 mM) for 3-24 h, and apoptosis was quantified by flow cytometry. The percentage of apoptotic cells (sub G0/G1 cell cycle phase) is represented. (D) Caspase-3 activation after 3-14 h of HF and/or HG incubation, and nuclei piknosis (detailed in a bright field) and cell loss in 14 h-stimulated cardiomyocytes. *p < 0.05 and **p < 0.01 vs. related control. †p < 0.05 and ††p < 0.01 vs. ZDF rats. [file 1475-2840-12-172-S2.pdf]

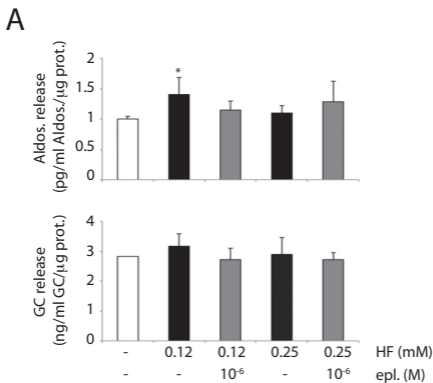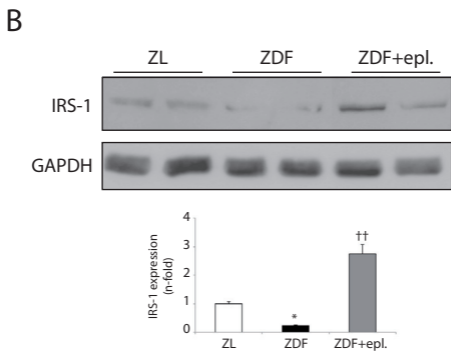

**Suppl. Figure 2.**

Supplement: Additional file 3: Figure S2 — (A) Aldosterone and glucocorticoids release from cardiomyocytes. Aldosterone and glucocorticoids (GC) were measured in cultured media after 12 h HF and HF+eplerenone incubations. (B) Cardiac IRS-1 expression. Total IRS-1 levels (~130 kDa) were detected in rat myocardia. *p < 0.05 vs. related control. ††p < 0.01 vs. ZDF rats. [file 1475-2840-12-172-S3.pdf]
